# Supplementary figures and images for: Causality Analysis for COVID-19 among Countries Using Effective Transfer Entropy
Source: Entropy (Basel). 2022 Aug 13;24(8):1115. doi: 10.3390/e24081115 (PMC9407067; doi:10.3390/e24081115)

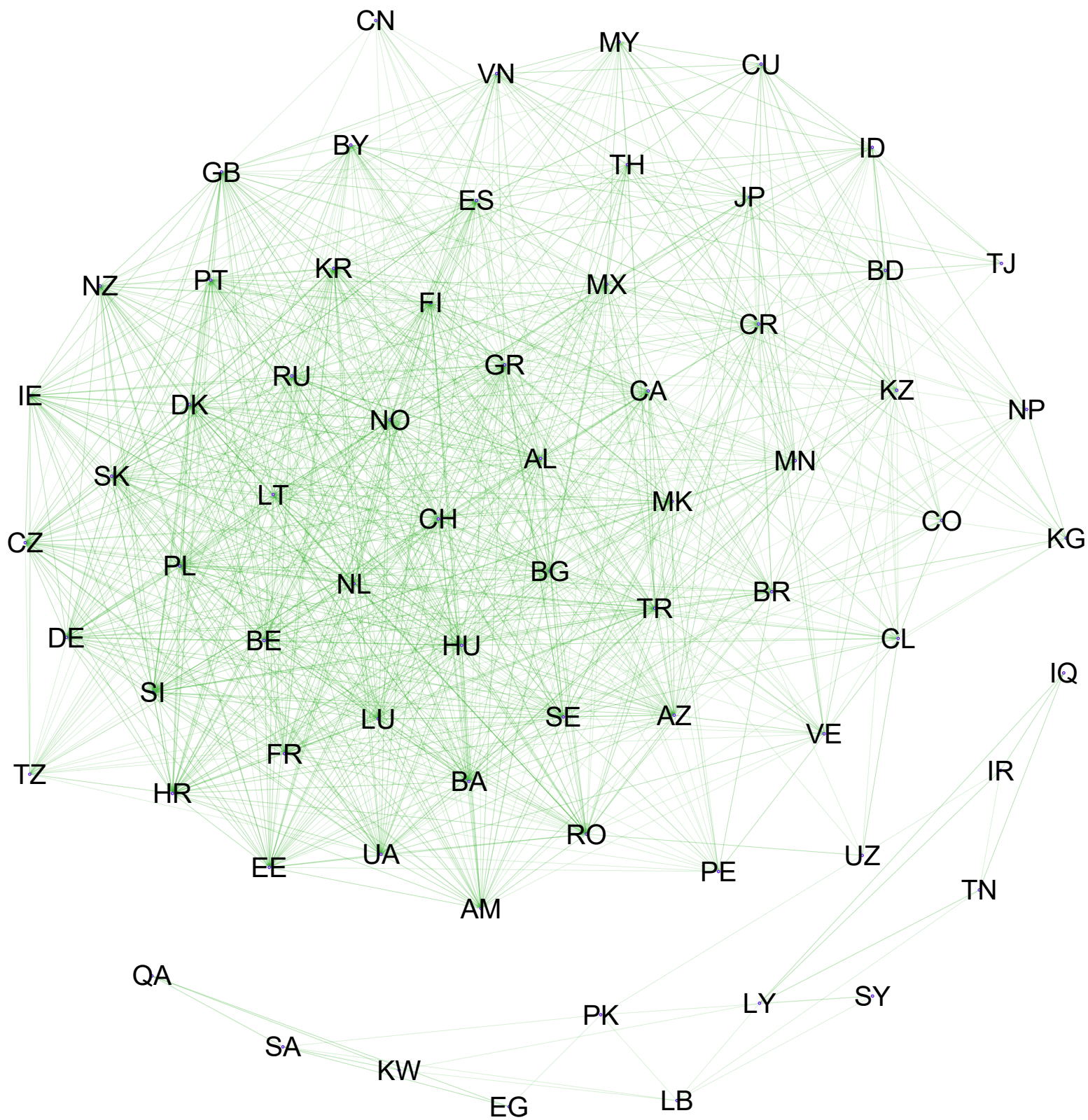

Supplement: Supplementary file 1 [file entropy-24-01115-s001.zip › Figure 1. Causality network.pdf]
